# Feasibility of Bioelectrical Impedance Spectroscopy Measurement before and after Thoracentesis

**DOI:** 10.1155/2015/810797

**Published:** 2015-03-11

**Authors:** Matthias Daniel Zink, Sören Weyer, Karolin Pauly, Andreas Napp, Michael Dreher, Steffen Leonhardt, Nikolaus Marx, Patrick Schauerte, Karl Mischke

**Affiliations:** ^1^Department of Cardiology, Pneumology, Angiology and Intensive Care Medicine, University Hospital, RWTH Aachen University, Pauwelsstraße 30, 52074 Aachen, Germany; ^2^Philips Chair for Medical Information Technology, RWTH Aachen University, Schinkelstraße 2, 52062 Aachen, Germany

## Abstract

*Background.* Bioelectrical impedance spectroscopy is applied to measure changes in tissue composition. The aim of this study was to evaluate its feasibility in measuring the fluid shift after thoracentesis in patients with pleural effusion.* Methods.* 45 participants (21 with pleural effusion and 24 healthy subjects) were included. Bioelectrical impedance was analyzed for “Transthoracic,” “Foot to Foot,” “Foot to Hand,” and “Hand to Hand” vectors in low and high frequency domain before and after thoracentesis. Healthy subjects were measured at a single time point.* Results.* The mean volume of removed pleural effusion was 1169 ± 513 mL. The “Foot to Foot,” “Hand to Hand,” and “Foot to Hand” vector indicated a trend for increased bioelectrical impedance after thoracentesis. Values for the low frequency domain in the “Transthoracic” vector increased significantly (*P* < 0.001). A moderate correlation was observed between the amount of removed fluid and impedance change in the low frequency domain using the “Foot to Hand” vector (*r* = −0.7).* Conclusion.* Bioelectrical impedance changes in correlation with the thoracic fluid level. It was feasible to monitor significant fluid shifts and loss after thoracentesis in the “Transthoracic” vector by means of bioelectrical impedance spectroscopy. The trial is registered with Registration Numbers IRB EK206/11 and NCT01778270.

## 1. Introduction

The etiology of pleural effusions (PE) can be local or systemic with more than 50 known causes [1, 2]. A PE is defined as an increase in the amount of fluid in the pleural space due to local or systemic causes; in particular congestive heart failure, pneumonia, and cancer are common causes of PE [3]. As a result, it can compromise respiratory function resulting in dyspnea, reduced exercise tolerance, and chest pain [4].

The diagnosis of a PE is usually based on clinical examination, ultrasound, and radiography. Ultrasound, as the gold standard, is able to quantify and characterize the PE [5]. Nevertheless, an experienced physician is required to quantify the amount of fluid. Thoracentesis is performed for diagnostic or therapeutic purposes [6]. Therapeutic thoracentesis is mostly applied in case of ineffective drug therapy or clinical symptoms such as dyspnea do not resolve fast enough [3].

**Figure 1:**
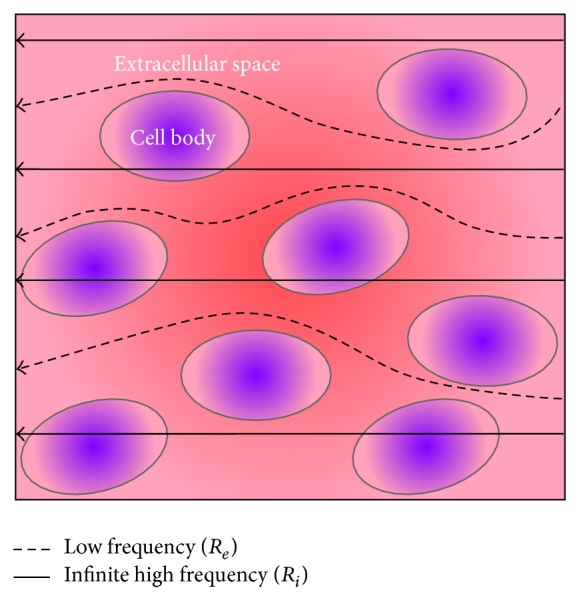
A model of the frequency-dependent electrical behavior of body tissue. At infinite high frequencies, the current passes more or less straight through all kinds of tissue; at low frequencies, the current avoids the cells.

Bioelectrical impedance spectroscopy (BIS) is based on the frequency-dependent electrical properties of body tissues. An electric current with high frequency passes more or less straight through most biological tissue; if the electric current is applied with a low frequency, it prefers the extracellular fluid filled space [7] (Figure 1, Table 2). An increase in the amount of fluid in the measured compartments decreases the bioelectrical impedance. BIS measurements have been used for monitoring hemodialysis [8], body fat analysis [9], or characterizing tissue properties [10, 11]. Although the use of bioelectrical impedance might point out the underlying disease, for instance cardiac or noncardiac cause of dyspnea, strong data supporting this hypothesis are still lacking [12].

The aim of this study was to evaluate the feasibility of different frequency domains and vectors for bioelectrical impedance spectroscopy to detect a fluid shift after thoracentesis in pleural effusion.

## 2. Materials and Methods

The study was performed from January 2012 to March 2013 at the Department of Cardiology, Pneumology, Angiology and Intensive Care Medicine, University Hospital RWTH Aachen, Germany. The trial was approved by the Ethics Committee of the University Hospital Aachen (Registration Number: EK206/11, date: 28 September 2011; Clinical Trials gov.: NCT01778270) and met current legal requirements (German medical devices act and Code of Medical Ethics) as well as ethical principles with their origin in the Declaration of Helsinki and Good Clinical Practice guidelines.

All participating patients signed informed consent voluntarily and were enrolled according to the following inclusion criteria: presence of symptomatic pleural effusion with an indication for pleural drainage and at least 18 years of age. Exclusion criteria were pregnancy or lactation, implanted device for electrical diagnosis or therapy, and mentally incapacitated patients.

Initially, we evaluated 26 patients; however, 5 were excluded due to the presence of only a small amount of pleural effusion (<500 mL). 24 healthy subjects were included as a control group. Informed consent was obtained from all participants.

Thoracentesis was performed by a physician of the Department of Pneumology if a minimum of 500 mL of fluid was drainable using the standard technique assisted by ultrasound. Thoracentesis was stopped if the patient felt discomfort or after 2000 mL was drained. The dependent variable was the impedance measured in lying position with the upper body elevated at an angel of 45 degrees before and after thoracentesis, and the independent variables were the measuring vector, frequency domain, and the drained volume of pleural effusion.

A measuring cart was built equipped with an “IntelliVue MX800 Patient Monitor” (Koninklijke Philips N. V., Amsterdam, Netherlands). The electrical integrity EN IEC 60601-1 was approved by the “Verband Deutscher Elektrotechnik” (VDE, Verband der Elektrotechnik, Elektronik Informationstechnik e.V., Frankfurt, Germany). For data acquisition an electronic case report form was created in OpenClinica (OpenClinica, LLC, Waltham, MA, USA) using the clinical data interchange standards consortium operational data model (CDISC, Austin, TX, USA).

### 2.1. Bioelectrical Impedance Spectroscopy

Biological tissue shows a frequency-dependent behavior to an applied alternating electrical signal. Under an alternating electrical excitation, biological tissue has a complex bioelectrical impedance which depends on its composition and frequency of the applied electrical current. Complex quantities can be represented by the real part of impedance, the resistance, and the imaginary part, the reactance.

The frequency response of the bioelectrical impedance of the biological tissues can provide information about its physiological composition. For example, impedance measurement (impedance tomography) during the aspiration of pleural effusion and the infusion of normal saline showed significant differences [13]. Different methods of impedance measurements are sensitive for the measurement of thoracic fluid shift [14, 15].

In this work a method called bioelectrical impedance spectroscopy was used. Bioelectrical impedance spectroscopy estimates the real and imaginary part of the electrical impedance over a wide frequency range. Low frequency current is mainly conducted by the extracellular and fluid filled space, whereas high frequency current is conducted more or less straight through all kinds of tissue (Figure 1, Table 2). A description of the electrical model and the mixture theory model was published by de Lorenzo et al. [16]. Fitting the measured impedance data to one of those models, the so-called Cole model, the resistance at zero and at infinite frequency can be extrapolated, relating to the extracellular resistances (*R*_*e*_) and the total resistances, respectively [17, 18]. The total resistance is a parallel combination of the extra- and intracellular resistance; thus the intracellular resistance (*R*_*i*_) can also be calculated.

A commercial instrument (SFB7; ImpediMed LTd., Brisbane, Australia) was used in this study to measure the impedance of the segments. All measurements were performed in accordance with the manufacturer's instruction manual.

**Figure 2:**
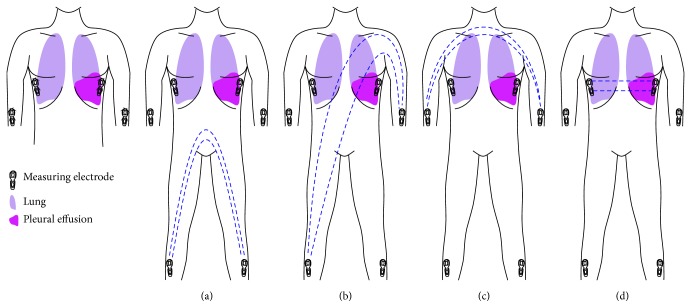
Measured vectors of bioelectrical impedance spectroscopy: (a) “Foot to Foot” (*F*); (b) “Foot to Hand” (*H*); (c) “Hand to Hand” (*B*); and (d) “Transthoracic” (*T*).

The measured voltage divided by the applied current is the bioelectrical impedance of the vector. In this paper, the feasibility of four measuring vectors (Figure 2, Table 3) and the extrapolation of the measured signal to a low frequency domain (*R*_*e*_) and infinite high frequency domain (*R*_*i*_) to determine presence of PE and fluid shift after thoracentesis was investigated.

### 2.2. Statistical Analysis

Statistical analysis was performed with IBM SPSS Statistics version 21 (IBM Corporation 1994, 2013) and MedCalc Statistical Software version 12.7.5 (MedCalc Software bvba, Ostend, Belgium; http://www.medcalc.org; 2014). Data are expressed as mean ± 95% confidence interval (CI) or ± standard deviation (SD). The Mann-Whitney *U* test for unpaired data or the paired *t*-test according to the data distribution was used for the comparisons. For the correlation of drained PE and bioelectrical impedance the linear regression was calculated. *P* < 0.01 was considered as statistically significant.

## 3. Results

The study was performed from January 2012 to March 2013. Nine (43%) of the measured patients were male (Table 1). The mean ± standard deviation (± SD) age was 69 ± 11 years. The baseline bodyweight was 76 ± 18 kg, body mass index was 25.9 ± 5.7 kg/m^2^, and the mean heart rate was 74 ± 18 beats per minute. Eleven (52%) punctuations were performed on the left side of the thorax. On average, 1169 ± 513 mL fluid was extracted. In 17 patients (81%), the PE was a transudate. Five patients were excluded from the study because the amount of PE did not warrant thoracentesis. No complications related to thoracentesis were observed.

**Figure 3:**
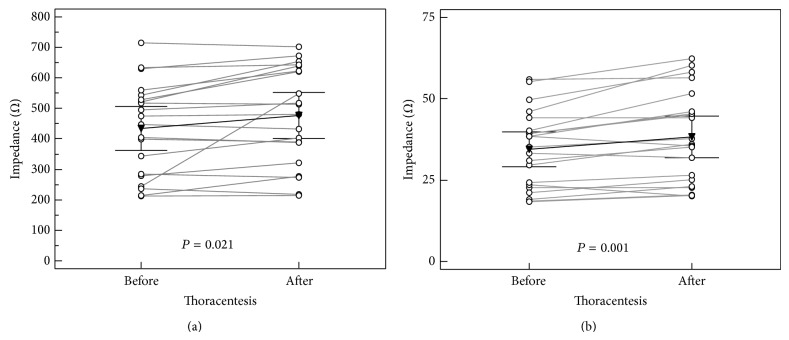
Effect of thoracentesis in the low frequency domain (*R*_*e*_): (a) impedances using the “Foot to Hand” vector (before 437 (95% CI 369–505) Ω; after 477 (95% CI 402–552) Ω; *P* = 0.021); (b) impedances using the “Transthoracic” vector (before 34.46 (95% CI 29.08–39.84) Ω; after 38.28 (95% CI 31.85–44.71) Ω; *P* = 0.001).

No relevant changes in impedance were observed after thoracentesis in “Foot to Foot” (*F*) vector measurements (Table 4). The “Hand to Hand” (*H*) (*H*_*R*_*e*_, before 472 (95% CI 411–533) Ω; after 502 (95% CI 437–565) Ω; *P* = 0.055) and the “Foot to Hand” (*B*) (*B*_*R*_*e*_; before 437 (95% CI 369–505) Ω; after 477 (95% CI 402–552) Ω; *P* = 0.021, Figure 3(a), Table 4) measurements showed a trend towards increased impedance in the low frequency domain after thoracentesis. The “Transthoracic” (*T*) vector showed a significant increase in impedance (*T*_*R*_*e*_; before 34.46 (95% CI 29.08–39.84) Ω; after 38.28 (95% CI 31.85–44.71) Ω; *P* = 0.001, Figure 3(b), Table 4) in the low frequency domain.

Compared to the control group, impedances in the low frequency domain measured using the “Foot to Foot” (*F*_*R*_*e*_, *P* = 0.19), “Hand to Hand” (*H*_*R*_*e*_, *P* = 0.1), and “Foot to Hand” (*B*_*R*_*e*_; *P* = 0.076) vectors indicated a trend towards lower values in patients before and after thoracentesis. Use of the “Transthoracic” vector resulted in a significantly lower impedance in patients before and after thoracentesis compared to the control group (*T*_*R*_*e*_; *P* = 0.001). For the high frequency domain we recorded no changes using the “Transthoracic” vector (*T*_*R*_*i*_; *P* = 0.3) but a significant bioelectrical impedance difference of the measurement before and after thoracentesis compared to the control group (Table 4) when applying the “Foot to Foot” (*F*_*R*_*i*_; *P* < 0.001), “Hand to Hand” (*H*_*R*_*i*_; *P* < 0.001), and “Foot to Hand” (*B*_*R*_*i*_; *P* < 0.001) vector.

**Figure 4:**
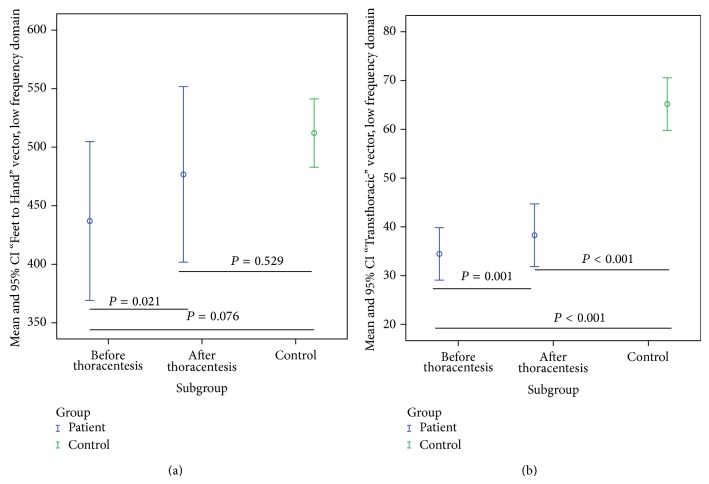
Impedances using the low frequency domain in patients before and after thoracentesis and in the control group: (a) using the “Foot to Hand” vector before (437 (369–505) Ω) and after thoracentesis (477 (95% CI 402–552) Ω), control group (512 (95% CI 483–541) Ω); (b) using the “Transthoracic” vector before (34.46 (29.08–39.84) Ω) and after thoracentesis (95% CI 38.28 (95% CI 31.85–44.71) Ω), control group (65.18 (95% CI 59.8–70.56) Ω).

The differences in bioelectrical impedance after thoracentesis compared to the control group for the low frequency domain using the “Foot to Foot” (*F*_*R*_*e*_; *P* = 0.3), “Hand to Hand” (*H*_*R*_*i*_; *P* = 0.38), and “Foot to Hand” (*B*_*R*_*e*_, *P* = 0.53, Figure 4(a), Table 4) vectors were not significant. However, using the “Transthoracic” (*T*_*R*_*e*_, *P* < 0.001, Figure 4(b), Table 4) vector resulted in significantly lower impedance measurements in patients after thoracentesis than in the control group.

**Figure 5:**
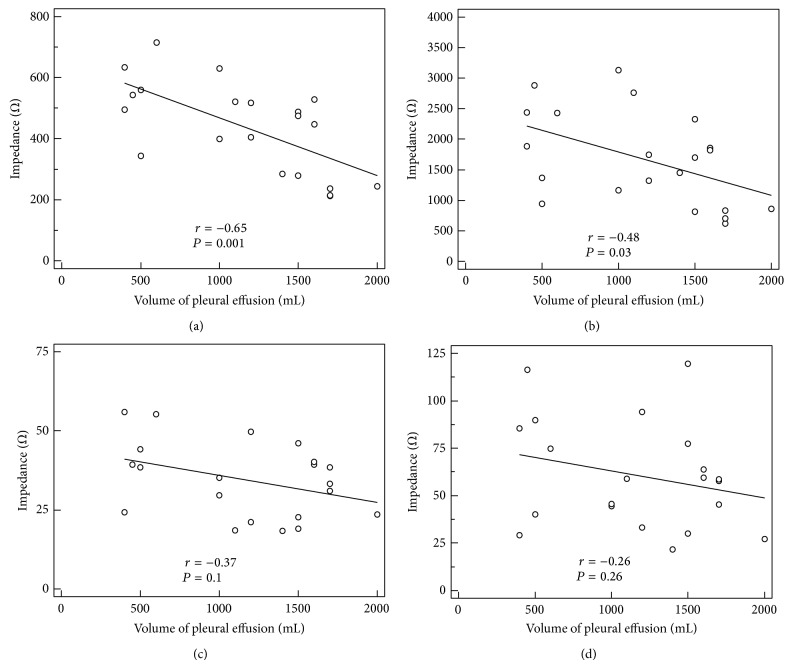
Correlation between PE and impedances using different measuring vectors: (a) “Foot to Hand” vector in the low frequency domain (*B*_*R*_*e*_; *r* = −0.65; *P* = 0.001; CI 95% −0.85 to −0.31), (b) “Foot to Hand” vector in the high frequency domain (*B*_*R*_*i*_; *r* = −0.48; *P* = 0.03; CI 95% −0.76 to  –0.06), (c) “Transthoracic” vector in the low frequency domain (*T*_*R*_*e*_; *r* = −0.37; *P* = 0.1; CI 95% −0.69 to 0.07), and (d) “Transthoracic” vector in the high frequency domain (*T*_*R*_*i*_; *r* = −0.26; *P* = 0.26; CI 95% −0.62 to 0.2).

A significant and moderate correlation was observed between the amount of drainable PE and the measured biological impedance when using the “Foot to Hand” vector for the low (*B*_*R*_*e*_; *r* = −0.65; *P* = 0.001; Figure 5(a)) frequency domain. The correlations between extracted PE and impedance measurements using the “Foot to Hand” vector in the high frequency domain (*B*_*R*_*i*_; *r* = −0.48; *P* = 0.03; Figure 5(b)) as well as the “Transthoracic” vector in the low (*T*_*R*_*e*_; *r* = −0.37; *P* = 0.1; Figure 5(c)) and high frequency domains (*T*_*R*_*i*_; *r* = −0.26; *P* = 0.26; Figure 5(d)) were poor.

## 4. Discussion

The present study demonstrates that changes in thoracic fluid content due to thoracentesis in pleural effusion resulted in an increase in bioelectrical impedance. The results furthermore show the potential of different measuring vectors and frequency domains for bioelectrical impedance measurement to determine fluid shifts in the pleural space.

Diagnosis and therapy control of PE are currently mainly performed by ultrasound and chest X-ray [19]. Although ultrasound is fast and inexpensive, it relies on the clinician's expertise, and X-ray is associated with radiation exposure. For this reason, a continuous, investigator-independent and safe method for noninvasive PE surveillance might help physicians to diagnose and monitor PE. Prior studies demonstrated correlations between Transthoracic impedance and thoracic fluid changes [20–23], but its clinical application remains limited due to a wide variability in impedance values in normal and pathological settings [14] as well as the influence of body position and electrode placement [20]. Nowadays, commercially available systems allow easy-to-use, noninvasive, safe, and physician-independent reproducible analysis of the fluid content in different body compartments [24].

### 4.1. Frequency Domain and Measuring Vector

Depending on the measuring vector a pleural effusion represents different proportions of the measured compartment (Figures 2(a)–2(d)). A pleural effusion would be expected to have a high impact on impedance when using the “Transthoracic” vector due to its high percentage of the targeted body volume. On the other hand, the “Transthoracic” vector represents the smallest targeted body volume (more or less only part of the thoracic cavity), so impedance measurements might be more affected by the localization of the pleural effusion than other vectors that target a larger measuring volume.

In our study, significant increases in bioelectrical impedance were observed after thoracentesis using the low frequency domain, whereas no significant changes after thoracentesis using the high frequency domain could be observed. The PE makes up a relevant volume of the thoracic volume, and thus vectors that focus on the thoracic volume, especially the “Transthoracic” vector, would be expected to show a higher increase in bioelectrical impedance after thoracentesis. Indeed, significant increases in bioelectrical impedance after thoracentesis were observed using the “Transthoracic” vector, and a trend was observed using the “Foot to Hand” and “Hand to Hand” vectors. As the “Foot to Foot” vector (Figure 2(a)) measures predominantly the lower body parts no change in bioelectrical impedance spectroscopy was seen after thoracentesis. Our results therefore confirm the theoretical considerations with regard to the performance of different vectors.

### 4.2. Identification of Pathological Situations

Due to a wide range of pathological and normal values in preceding studies [14, 20] Transthoracic bioelectrical impedance measurement did not seem applicable for clinical use. In our study, the use of different frequency domains and vectors for measurement suggests that the low frequency domains in combination with the “Foot to Hand” and even better the “Transthoracic” vector have a higher potential to identify pathological situations. However, this would need to be confirmed in further studies.

### 4.3. Pleural Effusion Volume Correlation with the Bioelectrical Impedance Change

Using the “Foot to Hand” vector in the low frequency domain we observed a moderate correlation between the volume of the extracted pleural effusion and the change in bioelectrical impedance. Interestingly and in contrast to its ability to detect significant changes of impedance before and after thoracentesis, use of the “Transthoracic” vector offered a poor correlation of bioelectrical impedance increase to the extracted volume. This finding might be related to the smaller targeting volume of the “Transthoracic” vector, and part of the drainable PE might have been “missed.”

Additionally, measurements with the “Transthoracic” vector might be more influenced by fluid shifts and posture changes, as seen in other studies, demonstrated by the wide range of pathological and normal values [20]. Therefore, the “Foot to Hand” vector in the low frequency domain might help to correlate the recorded values with the amount of drainable PE.

Different systems for thoracic impedance measurement are in focus of research [15, 22, 23] recording significant results in fluid surveillance but suffering of similar pitfalls like electrode placement, posture influence, or calculation errors by their mathematical algorithm [23]. Due to their typical electrode position placed on or next to the chest, the resulting target volumes might be too small to include the entire fluid amount. Additionally, posture change might even aggravate this problem. These problems might in part be overcome by using the “Foot to Hand” in combination with the “Transthoracic” vector.

There is a lack of standardization for the frequency used for bioelectrical impedance measurement. A rather large frequency range (5 kHz to 1 MHz) might be most suitable for BIS as indicated by the Cole model [25, 26]. We used both, an extrapolated low frequency to zero (*R*_*e*_) and an extrapolated high to infinite (*R*_*i*_) frequency, and the low frequency domain allowed detection of fluid shifts after thoracentesis.

### 4.4. Limitations

The measured data were fitted to the Cole model which represents different conduction properties of body tissue [27]. Therefore, the calculated values do not necessarily result from changes by thoracentesis but could also be influenced by other changes in the intra- or extracellular fluid. To exclude these factors, the measurements were performed before and after thoracentesis and in a predefined posture position. No additional therapies like administration of diuretics or fluid intake were performed between these two measurements. The second limitation was the minimal amount of 500 mL of drained PE. Therefore, we do not know whether smaller amounts of PE would have resulted in significant impedance differences between patients with PE and probands in the control group. In addition, we did not evaluate the ability of bioelectrical impedance spectroscopy to differentiate between pleural effusion and other causes of fluid excess such as pulmonary edema, and no cut-off values were established for clinical use.

## 5. Conclusion

In conclusion, a significant increase in bioelectrical impedance was observed using the low frequency domain for the “Transthoracic” vector after thoracentesis. There was a moderate correlation between the amount of removed PE and the change in BIS using the “Foot to Hand” vector in the low frequency domain. The present study demonstrates the feasibility of measuring fluid shifts by bioelectrical impedance spectroscopy in thoracentesis and might be used as an adjunct diagnostic tool to evaluate pleural effusions and monitor patients after thoracentesis. BIS measurement was safe, noninvasive, and easy to handle. However, this has to be seen in light of ultrasound technology, which is easy to use and usually widely available, so BIS technology could be interesting to use in addition to standard ultrasound. For instance, integration of BIS into standard monitoring in intensive or intermediate care units using electrodes that are already used to monitor ECG and respiration rate might allow earlier detection of fluid changes.

## Figures and Tables

**Table 1:** Baseline data.

Baseline data		

	Patient data	Control
	Mean ± SD/percentage	Mean ± SD/percentage
	*n* = 21	*n* = 25

Thoracentesis performed	21	0
Age	69 ± 11	26 ± 3
Sex		
Female [%]	57	36
Height [cm]	171 ± 8	179 ± 8
Weight [kg]	76 ± 18	76 ± 18
Body mass index [kg/m^2^]	25.9 ± 5.7	23.5 ± 3.6

Organ failure		
Heart [%]	81	
Reason for pleural effusion		
Cardiac decompensation [%]	86	
Type of pleural effusion		
Transudate [%]	81	
Left ventricular ejection fraction [%]	49 ± 16	
Blood pressure systolic/diastolic [mmHg]	121 ± 17/71 ± 9	
Heart rate [beats per minute]	74 ± 18	
NT-proBNP [pg/mg]	3948 ± 5947	
Oxygen saturation [%]	96 ± 2	
Pleural effusion [mL]	1169 ± 513	
Pleural effusion side		
Left [%]	52	

**Table 2:** Measured parameters.

Measured parameters of bioelectrical impedance spectroscopy		
Abbreviation	Unit	Description
*R*_*e*_	Ohm [Ω]	The impedance of the low frequency current path extrapolated to zero based on the Cole model for extracellular impedance

*R*_*i*_	Ohm [Ω]	The intracellular resistance refers to the resistance of the intracellular space only. It cannot be measured but computed from the resistance at high and low frequencies

**Table 3:** Vectors for bioelectrical impedance measurement.

Vectors for bioelectrical impedance measurement	
Abbreviation	Description
*F*	“Foot to Foot” vector: the pads were placed proximal and lateral to the ankle on the left and right leg

*H*	“Hand to Hand” vector: the pads were placed proximal and dorsal to the wrist on the left and right arm

*B*	“Foot to Hand” vector for whole body impedance with the pad placed proximal and lateral to the ankle on the left leg and the proximal and dorsal to the wrist of the left arm

*T*	“Transthoracic” vector measurement: the pads were placed in the 5th intercostal spaces in the left and right axillary lines

**Table 4:** Bioelectrical impedance results before and after thoracentesis (95% CI).

Vector	Parameters	Thoracic impedance					
		Before thoracentesis	After thoracentesis	Control	*P* value^*^	*P* value^**^	*P* value^***^
*F*	*R*_*e*_ [Ω]	397 (316–478)	430 (339–520)	422 (398–446)	0.133	0.185	0.301
	*R*_*i*_ [Ω]	1770 (1303–2236)	1783 (1378–2187)	697 (600–793)	0.536	<0.001	<0.001

*H*	*R*_*e*_ [Ω]	472 (411–533)	502 (437–567)	532 (499–565)	0.055	0.1	0.375
	*R*_*i*_ [Ω]	1742 (1338–2146)	1699 (1277–2121)	835 (748–922)	0.823	<0.001	0.001

*B*	*R*_*e*_ [Ω]	437 (369–505)	477 (402–552)	512 (483–541)	0.021	0.076	0.529
	*R*_*i*_ [Ω]	1670 (1323–2018)	1636 (1272–2001)	765 (696–835)	0.996	<0.001	<0.001

*T*	*R*_*e*_ [Ω]	34.46 (29.08–39.84)	38.28 (31.85–44.71)	65.18 (59.8–70.56)	0.001	<0.001	<0.001
	*R*_*i*_ [Ω]	60.57 (47.64–73.5)	62.11 (49.12–75.09)	69.21 (56.12–82.29)	0.705	0.331	0.382

^*^ *P* value between before and after thoracentesis.

^**^ *P* value between before thoracentesis and control group.

^***^ *P* value between after thoracentesis and control group.

*F*: “Foot to Foot” vector, *H*: “Hand to Hand” vector, *B*: “Foot to Hand” vector, *T*: “Transthoracic” vector, *R*_*e*_: extrapolated resistance of the low frequency to “0” as resistance of extracellular space, and *R*_*i*_: extrapolated resistance of the high frequency to infinite as resistance of intracellular space.

## Abbreviations

*B*: “Foot to Hand” impedance as body vector
BIS: Bioelectrical impedance spectroscopy
*F*: “Foot to Foot” impedance vector
*H*: “Hand to Hand” impedance vector
PE: Pleural effusion
*R*_*e*_: Extrapolated resistance of the low frequency to “0” as resistance of extracellular space
*R*_*i*_: Extrapolated resistance of the high frequency to infinite as resistance of intra- and extracellular space
*T*: “Transthoracic” impedance vector.
